# Design and Error Calibration of a Machine Vision-Based Laser 2D Tracking System

**DOI:** 10.3390/s26020570

**Published:** 2026-01-14

**Authors:** Dabao Lao, Xiaojian Wang, Tianqi Chen

**Affiliations:** School of Automation and Electrical Engineering, University of Science and Technology Beijing, Beijing 100083, China; wangxiaojian@xs.ustb.edu.cn (X.W.); chentianqi@xs.ustb.edu.cn (T.C.)

**Keywords:** laser tracker, machine vision, tracking, error calibration, singular value decomposition

## Abstract

A laser tracker is an essential tool in the field of precise geometric measurement. Its fundamental operating idea is a dual-axis rotating device that propels the laser beam to continuously align and measure the attitude of a collaborating target. Such systems provide numerous benefits, including a broad measuring range, high precision, outstanding real-time performance, and ease of use. To solve the issue of low beam recovery efficiency in typical laser trackers, this research offers a two-dimensional laser tracking system that incorporates a machine vision module. The system uses a unique off-axis optical design in which the distance measuring and laser tracking paths are independent, decreasing the system’s dependency on optical coaxiality and mechanical processing precision. A tracking head error calibration method based on singular value decomposition (SVD) is introduced, using optical axis point cloud data obtained from experiments on various components for geometric fitting. A complete prototype system was constructed and subjected to accuracy testing. Experimental results show that the proposed system achieves a relative positioning accuracy of less than 0.2 mm (spatial root mean square error (RMSE) = 0.189 mm) at the maximum working distance of 1.5 m, providing an effective solution for the design of high-precision laser tracking systems.

## 1. Introduction

Precision measurement instruments serve as essential platforms for realizing high-accuracy measurement technologies and are indispensable tools in modern scientific research and advanced engineering manufacturing processes [1,2]. Due to their superior measurement accuracy, quick response time, and strong spatial adaptability, laser trackers have become one of the most popular key devices in high-end precision measurement, particularly in contemporary industrial measurement scenarios that increasingly demand large-scale measurement capabilities, high spatial resolution, and real-time responsiveness [3,4].

Laser trackers typically work on the basis of polar coordinate measurement. Their fundamental components are a laser tracking head (laser, rangefinder, Position Sensitive Detector (PSD), and other optical components), a two-axis rotating device, a controller (communication mode, external interface), a host computer (running software), a cooperative target, and additional measuring accessories. These systems allow for high-precision target point placement in three-dimensional space through coordinated control and data fusion [5,6]. In order to achieve continuous tracking and stable capture of dynamic targets, the system continuously modifies the laser’s orientation during actual measurement processes in response to changes in the cooperative target sphere’s spatial position, also referred to as the Spherically Mounted Retroreflector (SMR). Commercial laser tracking systems are currently widely used in high-end manufacturing industries, including mold inspection, automotive production, and aerospace because they have attained micrometer-level measurement accuracy.

Laser tracking technology has been a part of engineering application research since the 1980s. Laser tracker systems and products have become more intelligent, multifunctional, and smaller due to ongoing developments in critical technologies including digital signal processing, precision mechanical control, and photoelectric measurement. Even though the polar coordinate measurement framework based on laser interferometric distance measurement and two-dimensional angle encoding has been widely adopted in the industry, a number of technical bottlenecks still exist in terms of practical deployment and usage, despite the fact that current laser tracker technologies have reached a relatively mature stage.

First, conventional laser tracker systems usually use high-precision biaxial rotary stages. The accuracy of azimuth (horizontal angle) and elevation angle measurements is directly impacted by mechanical backlash, encoder resolution limitations, and rotational axis installation errors, which limit the system’s overall stability and measurement accuracy [7]. Second, the laser beam path will be interrupted during dynamic measurements if the target sphere is obscured or lost. The continuity of measurement tasks and overall system efficiency are then significantly impacted by the time-consuming and labor-intensive procedure of completely re-initializing the guiding beam path required by conventional systems. Meanwhile, as authoritative standards for the performance evaluation and verification of laser trackers, ASME B89.4.19-2021 and ISO 10360-10:2021 clearly specify core requirements such as periodic calibration and geometric error detection of equipment [8,9]. Traditional systems struggle to balance compliance with these standards and beam break recovery efficiency, which further highlights the necessity of designing the off-axis optical structure and visual reconnection mechanism in this study.

Recent studies have looked into integrating computer vision technology to create innovative laser tracking systems with vision-assisted capability in order to address these problems. For instance, these systems can locate and identify the target sphere’s pose by integrating industrial cameras and image processing modules, allowing for quick recovery and path correction after beam interruptions [10]. The development of unified standards or widely used mature solutions is hindered by the significant challenges that such systems still face in real-world engineering applications, such as intricate calibration processes, inconsistent image recognition accuracy, and high demands on mechanical assembly precision.

## 2. Overall System Design

This study presents the design of an intelligent laser tracking system featuring rapid beam break recovery based on machine vision. The core architecture comprises two main components: a two-axis rotary stage and a tracking head.

**Figure 1:**
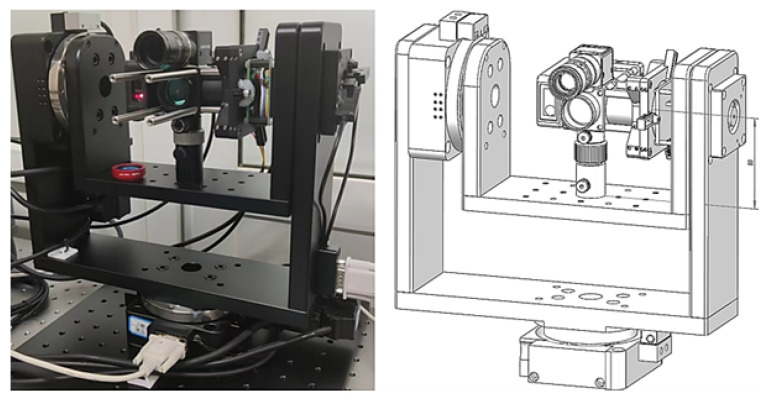
Schematic diagram of the proposed laser 2D tracking system.

As shown in Figure 1, this work proposes a novel machine vision-based design approach for a laser 2D tracking system to overcome these technical constraints. The proposed system introduces a novel off-axis optical arrangement where the vision recognition path and distance measurement path are configured separately. While preserving measurement accuracy, this configuration successfully reduces the system’s dependency on optical coaxiality and precise mechanical alignment. The technology assists with autonomous laser path guidance and dynamic attitude control by combining sophisticated image processing techniques with target identification algorithms to enable fast localization and reliable tracking of the SMR in image space.

Compared with traditional coaxial optical systems, the proposed design offers a more compact system architecture (with overall dimensions of approximately 300 mm × 300 mm × 310 mm). Notably, the system proposed in this study exhibits remarkable cost competitiveness compared with commercial laser trackers with equivalent dynamic tracking performance. The total development cost of the prototype system is approximately $25,000, covering all core hardware components (including the biaxial rotary stage, optical components of the tracking head, industrial camera, PSD sensor, Absolute Distance Meter (ADM) rangefinder, servo motor, and related mechanical structural parts), mechanical processing fees, and software development costs. In contrast, mainstream commercial devices on the market, represented by the API T3 and Leica AT960 laser trackers, can achieve a spatial root mean square error (RMSE) within 0.2 mm, and are equipped with dynamic target tracking and beam break recovery functions, with their market prices generally ranging from $80,000 to $150,000. This significant cost gap highlights the economic advantages of the proposed system, making it a more cost-effective option for budget-constrained scenarios such as laboratory research, small- and medium-sized industrial measurement, and customized precision detection.

To clarify the application positioning and characteristics of the proposed system, Table 1 presents a characteristic comparison with two mainstream commercial laser trackers (API T3 and Leica AT960, Wetzlar, Germany). The parameters of commercial products are comprehensively referenced from manufacturers’ technical manuals, while the indicators of the proposed system are derived from prototype experimental test results, focusing on the adaptability analysis in short-distance measurement scenarios.

With the goal of offering a practical and effective solution for high-robustness, high-precision, and long-range dynamic target tracking measurements, as well as providing technical support and theoretical underpinnings for the development of intelligent measurement systems, this paper thoroughly examines important issues like overall system architecture design, core functional module implementation, error modeling, and calibration methods.

**Figure 2:**
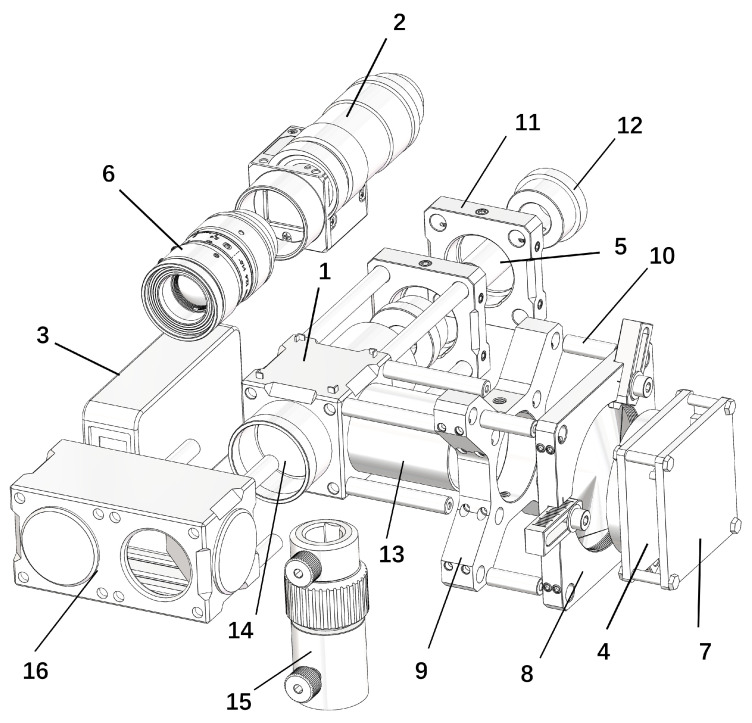
Exploded view of the tracking head structure of the proposed system.

A schematic illustration of the tracking head structure is shown in Figure 2. The tracking head adopts a composite optical-mechanical design, consisting of a laser source, a beam splitter, a PSD, an ADM, an industrial camera, and other essential cage optical components. The components corresponding to the labels in Figure 2 (along with their models and quantities) are detailed in Table 2.

The tracking head uses the center of the cubic beam splitter as its reference point, from which the instrument’s primary optical axis extends both forward and backward. The laser beam emitted from the laser source aligns precisely with this primary optical axis. The PSD sensor and ADM are located on either side of the cubic beam splitter. The distance measurement beam created by the ADM is aligned with the laser beam using an optical path regulator. The beam reflected back from the SMR is sent through the optical path regulator and cubic beam splitter, with the cubic beam splitter’s reflective surface directing a portion of this return beam towards the PSD sensor.

The system calculates the target sphere’s current spherical coordinates using the ADM and PSD sensor outputs. This information is then used to control the two-axis rotary stage, allowing for continuous target tracking. In the event of target loss, the system uses the industrial camera to search for the SMR while controlling the two-axis rotary stage to reorient toward the target, enabling quick beam path reacquisition.

**Figure 3:**
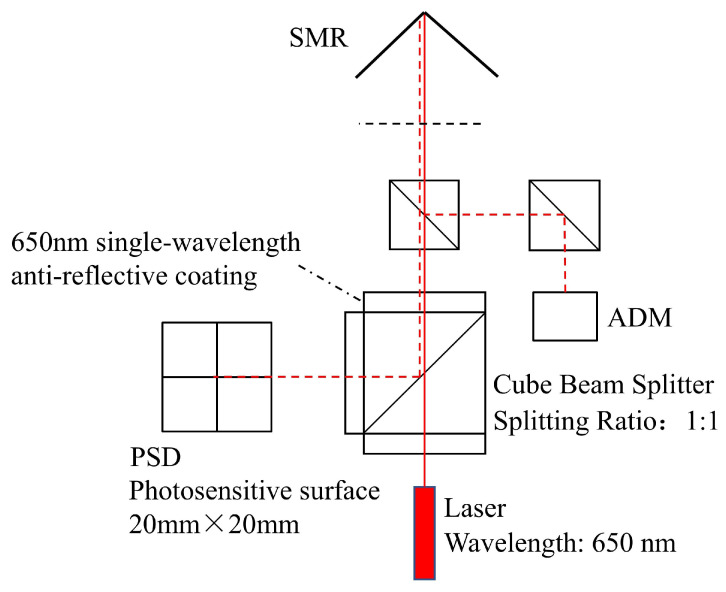
Schematic diagram of the optical path configuration of the proposed system.

Figure 3 shows the system’s optical path design. The laser beam created by the laser source travels immediately outside the instrument and is reflected back inside by the SMR target sphere. A portion of the returned beam is then routed to the PSD sensor via the cubic beam splitter located in the center of the optical path.

The system adopts an off-axis distance measurement scheme, dividing the optical path into two mutually independent sections: one for distance measurement and the other for target recognition. This design eliminates the need for overly complex optical coupling modules between the distance measurement optical path and the main optical path of the system. The distance measurement beam emitted by the ADM is projected onto the SMR target ball via a reflector, and the distance measurement is completed after the beam is reflected by the prism built into the target ball. The final measured distance value is regarded as the actual distance only after error compensation.

The cubic beam splitter used in this system features a 1:1 beam splitting ratio. Additionally, narrowband optical filters and lens shade tubes are installed on the external surfaces along the three optical transmission directions to ensure the PSD sensor receives sufficient light intensity for normal operation while effectively minimizing interference from ambient light. This design separates the tracking head from the biaxial rotary mechanism, allowing the laser beam to directly project outside the instrument and simplifying the overall system architecture.

**Figure 4:**
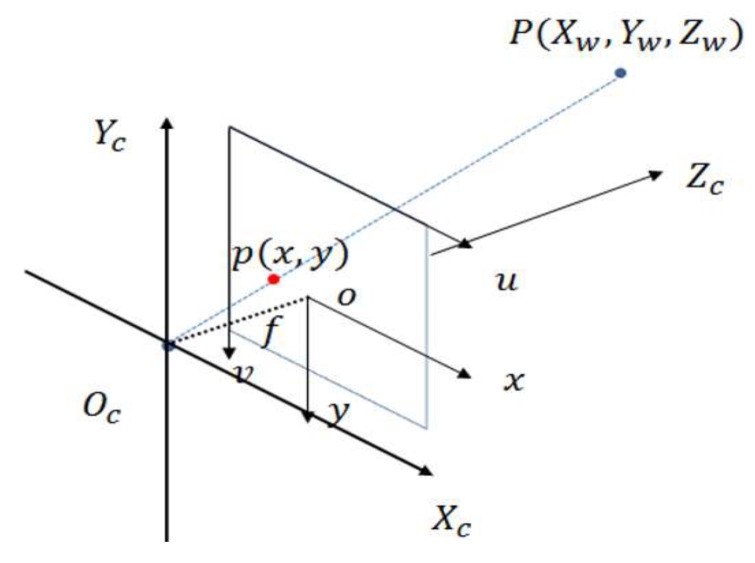
Schematic diagram of camera model and imaging coordinate systems.

Current commercial laser trackers (e.g., Leica AT960, API T3) generally adopt an architecture of “single optical path coaxial integration + camera-main optical axis binding”: On one hand, the laser, distance measurement module, and position detection components need to share the same main optical axis, relying on specialized optical coupling components to achieve optical path integration. This imposes extremely high requirements on the precision of mechanical assembly, which not only increases hardware configuration costs but also extends the system’s assembly and commissioning cycle; On the other hand, their visual auxiliary cameras need to adopt an coaxial layout, and through precise mechanical adjustment, the camera’s optical axis maintains a fixed spatial correspondence with the main optical axis, with the field of view center fully coinciding with the main optical axis direction, this calibration process is complex to operate, and if mechanical structure deviation occurs due to environmental disturbances in the later stage, it will directly affect the effectiveness of beam break recovery. In contrast, the proposed system adopts an “off-axis splitting + camera optical path decoupling” design, as illustrated in Figure 4. Specifically, two key design features are implemented as follows:

1. The laser and the ADM distance measurement module each maintain their own independent coaxial optical paths. These two paths achieve off-axis coordination through a cube beam splitter, eliminating the requirement for full-link optical path coaxiality and thereby significantly reducing the system’s dependence on high-precision mechanical assembly.
2. The optical path of the visual camera is completely independent from the main optical axis, with no need for rigid mechanical calibration or binding between them. Instead, laser beam guidance is accomplished solely by algorithmically matching the position of the SMR in the camera image with the pointing direction of the main optical axis.

This design not only simplifies the system’s assembly process and controls hardware costs but also avoids the environmental sensitivity issues caused by mechanical binding, improving the reliability of the beam break recovery function.

## 3. Error Calibration Method

### 3.1. Monocular Camera Calibration

In the novel laser tracker, the machine vision module is responsible for searching the target sphere in the field of view when the system is in a beam-interrupted state, and then deriving the world coordinate system of the target. Therefore, it is necessary to first understand the imaging principle of industrial cameras. As shown in Figure 4, four coordinate systems are involved in the figure: the world coordinate system ($O_{w}-X_{w}Y_{w}Z_{w}$, describing the camera position, unit: m), the camera coordinate system ($O_{c}-X_{c}Y_{c}Z_{c}$, with the optical center as the origin, unit: m), the image coordinate system (o−xy, with the optical center as the midpoint of the image, unit: mm), and the pixel coordinate system (o−uv, with the origin at the upper left corner of the image, unit: pixel). Among them, point *P* is a real point in the physical world, point *p* is the corresponding point in the image, and *f* is the camera focal length.

The world coordinate system, also known as the object coordinate system, is used to clearly represent and describe objectively existing objects. Assume that the coordinate of a point *P* in space in the world coordinate system is $\left(x_{w},y_{w},z_{w}\right)$, and its coordinate in the camera coordinate system is $\left(x_{c},y_{c},z_{c}\right)$. The two coordinates can be converted through rigid body transformation, which includes translation and rotation. The transformation relationship is as follows:

(1) $$\left[\begin{array}{c} x_{c}\\ y_{c}\\ z_{c}\\ 1 \end{array}\right]=\left[\begin{array}{cc} R & t\\ 0^{T} & 1 \end{array}\right]\left[\begin{array}{c} x_{w}\\ y_{w}\\ z_{w}\\ 1 \end{array}\right]$$

where $t={\left[t_{x},t_{y},t_{z}\right]}^{T}$ is the translation vector during the conversion of the two coordinate systems, and R is the rotation matrix (3×3 square matrix), whose expression is related to the sine and cosine values of the rotation angles in three directions:

(2) $$R=\left[\begin{array}{ccc} \cos\alpha \cos\beta & \cos\alpha \sin\beta \sin\gamma -\sin\alpha \cos\gamma & \cos\alpha \sin\beta \cos\gamma +\sin\alpha \sin\gamma \\ \sin\alpha \cos\beta & \sin\alpha \sin\beta \sin\gamma +\cos\alpha \cos\gamma & \sin\alpha \sin\beta \cos\gamma -\cos\alpha \sin\gamma \\ -\sin\beta & \cos\beta \sin\gamma & \cos\beta \cos\gamma \end{array}\right]$$

The camera coordinate system takes the optical center of the lens as the origin, the $Z_{c}$ axis extends outward along the optical axis direction, and the plane composed of the $X_{c}$ and $Y_{c}$ axes is translated from the physical imaging plane. The transformation from the camera coordinate system to the image coordinate system is a perspective projection transformation from 3D to 2D. According to the similar triangle theorem, the coordinate $\left(x_{c},y_{c},z_{c}\right)$ of point *P* in the camera coordinate system can be converted to the image coordinate system coordinate $\left(x^{\prime },y^{\prime }\right)$:

(3) $$z_{c}\left[\begin{array}{c} x^{\prime }\\ y^{\prime }\\ 1 \end{array}\right]=\left[\begin{array}{cccc} f & 0 & 0 & 0\\ 0 & f & 0 & 0\\ 0 & 0 & 1 & 0 \end{array}\right]\left[\begin{array}{c} x_{c}\\ y_{c}\\ z_{c}\\ 1 \end{array}\right]$$

The image coordinate system is established based on the camera photosensitive sensor and can be regarded as a part of the physical imaging plane. An image is essentially an array of pixel gray values. The pixel coordinate system defines the upper left corner of the image as the origin, and *u* and *v* represent the row and column positions of a point in the image (unit: pixel), respectively. The image coordinate system and the pixel coordinate system can be converted to each other through translation and scaling transformation: the origin is shifted from the center of the imaging surface to the upper left corner of the image, and the unit is converted from mm to pixel. The conversion relationship is:

(4) $$\left[\begin{array}{c} u\\ v\\ 1 \end{array}\right]=\left[\begin{array}{ccc} \frac{1}{dx} & 0 & u_{0}\\ 0 & \frac{1}{dy} & v_{0}\\ 0 & 0 & 1 \end{array}\right]\left[\begin{array}{c} x^{\prime }\\ y^{\prime }\\ 1 \end{array}\right]$$

where dx and dy represent the millimeter size of a single pixel on the camera photosensitive surface in the horizontal and vertical directions, and $\left(u_{0},v_{0}\right)$ is the pixel coordinate of the image center when the origin is at the upper left corner. By combining the above coordinate transformation formulas, the mathematical model of camera imaging can be obtained:

(5) $$z_{c}\left[\begin{array}{c} u\\ v\\ 1 \end{array}\right]=\left[\begin{array}{cccc} f_{x} & 0 & u_{0} & 0\\ 0 & f_{y} & v_{0} & 0\\ 0 & 0 & 1 & 0 \end{array}\right]\left[\begin{array}{cc} R & t\\ 0^{T} & 1 \end{array}\right]\left[\begin{array}{c} x_{w}\\ y_{w}\\ z_{w}\\ 1 \end{array}\right]$$

where $f_{x}=\frac{f}{dx}$, $f_{y}=\frac{f}{dy}$, $\left[\begin{array}{cccc} f_{x} & 0 & u_{0} & 0\\ 0 & f_{y} & v_{0} & 0\\ 0 & 0 & 1 & 0 \end{array}\right]$ is the camera intrinsic parameter matrix, and $\left[\begin{array}{cc} R & t\\ 0^{T} & 1 \end{array}\right]$ is the camera extrinsic parameter matrix.

However, due to manufacturing process variations, inconsistencies in magnification between the lens center and edges result in image distortion [11]. Distortion is an inherent characteristic of optical systems. Although it does not affect image sharpness, it does cause geometric deformation of the captured images and requires algorithmic correction. The main types of distortion include the following: radial distortion, which intensifies with increasing distance from the optical axis, with minimal distortion at the center and either outward expansion (barrel distortion) or inward contraction (pincushion distortion) at the image edges; tangential distortion, caused by non-parallel assembly between the lens group and the image plane, resulting in inclined imaging of light rays; and thin prism distortion, which arises from material imperfections or assembly errors in the lens, causing asymmetric lateral displacement of light rays. Although optimized manufacturing processes can reduce these distortions, they cannot be completely eliminated, necessitating a subsequent calibration-based correction.

This study adopts Zhang’s calibration method, utilizing multiple images of a checkerboard calibration target captured from different poses to solve the intrinsic parameters of the camera through the Camera Calibration Toolbox in MATLAB R2022b. The selection of this method is mainly based on three core considerations: first, it only requires a planar checkerboard to complete the calibration without the need for high-precision 3D calibration objects, which significantly reduces hardware costs and operational complexity, and is suitable for rapid calibration scenarios in laboratories and industrial fields; second, it can simultaneously solve the camera’s intrinsic parameters (focal length, principal point coordinates, distortion coefficients) and extrinsic parameters (rotation matrix, translation vector), with a significant correction effect on lens radial and tangential distortions, which is consistent with the high-precision measurement requirements of this system; third, the algorithm has strong robustness, excellent anti-noise ability and data redundancy fault tolerance, and has been widely integrated into mainstream toolkits such as OpenCV and MATLAB, facilitating engineering implementation and secondary development [12]. Assuming a spatial coordinate system exists on the checkerboard, the coordinates of a corner point A on the board can be expressed as $\left(x_{a},y_{a},z_{a}\right)$. After capturing the calibration board image with the camera, corner point A corresponds to a pixel point a in the image, whose coordinates are $\left(u_{a},v_{a}\right)$. According to (5), the transformation relationship between these coordinate systems can be expressed as follows:

(6) $$s\left[\begin{array}{c} u_{a}\\ v_{a}\\ 1 \end{array}\right]=\left[\begin{array}{cccc} f_{x} & 0 & u_{0} & 0\\ 0 & f_{y} & v_{0} & 0\\ 0 & 0 & 1 & 0 \end{array}\right]\left[\begin{array}{cc} R & t\\ 0^{T} & 1 \end{array}\right]\left[\begin{array}{c} x_{a}\\ y_{a}\\ z_{a}\\ 1 \end{array}\right]=M_{1}\left[\begin{array}{cccc} r_{1} & r_{2} & r_{3} & t \end{array}\right]\left[\begin{array}{c} x_{a}\\ y_{a}\\ z_{a}\\ 1 \end{array}\right]$$

where *s* is a scale factor and $M_{1}$ and [$r_{1}$, $r_{2}$, $r_{3}$, $r_{4}$, t] are matrices related to the intrinsic parameters of the camera. Since all checkerboard corner points lie on the same plane, when the object coordinate system is established on this plane, the value of $z_{a}$ becomes zero. Consequently, (6) simplifies to:

(7) uaνa1=M1Sr1r2r3txaya01=M1Sr1r2txaya1

Leaving out the Z-axis value, the transformation from ($x_{a}$, $y_{a}$, 0) to ($u_{a}$, $v_{a}$) in (7) can be regarded as a two-dimensional projective transformation, characterized by homography. The term [*s*, $M_{1}$, $r_{1}$, $r_{2}$, t] defines the homography matrix $H_{r}$, which is a 3 × 3 matrix that characterizes the intrinsic properties of the camera and is independent of external conditions. When both sides of (7) are expressed in homogeneous coordinates, proportional scaling does not affect the equality, resulting in $H_{r}$ containing eight degrees of freedom. The homography matrix $H_{r}$ and the constraint can be rewritten as:

(8) $$\left\{\begin{array}{c} H_{r}=\left[\begin{array}{ccc} h_{11} & h_{12} & h_{13}\\ h_{21} & h_{22} & h_{23}\\ h_{31} & h_{32} & h_{33} \end{array}\right]\\ \sum_{i=1}^{3}\sum_{j=1}^{3}h_{ij}^{2}=1 \end{array}\right.$$

By substituting the reformulated homography matrix into (7) and simplifying, the following equation set is obtained:

(9) $$\left\{\begin{array}{c} h_{11}x_{a}+h_{12}y_{a}+h_{13}-h_{31}x_{a}u_{a}-h_{32}y_{a}u_{a}-h_{33}u_{a}=0\\ h_{21}x_{a}+h_{22}y_{a}+h_{23}-h_{31}x_{a}v_{a}-h_{32}y_{a}v_{a}-h_{33}v_{a}=0 \end{array}\right.$$

As shown in (8) and (9), solving the nine unknown parameters in $H_{r}$ requires at least nine independent equations. Therefore, at least four corner points from the checkerboard must be substituted into (9) to generate eight linear equations. By combining these with the constraint equation, a solvable equation system can be formed. The homography matrix is then solved using optimization algorithms such as Singular Value Decomposition (SVD) and the Levenberg–Marquardt algorithm.

In this study, camera calibration was conducted using the built-in Camera Calibration Toolbox in MATLAB. A custom-designed aluminum oxide optical calibration board was used as the target. Twenty images of the checkerboard from different camera poses were captured. After corner detection, several images with high similarity or large corner detection errors were discarded. The final calibration results are presented in Table 3.

### 3.2. Optical Axis Calibration of the Tracking Head

Due to manufacturing imperfections, the laser beam emitted from the laser source is not perfectly perpendicular to the exit surface but is instead projected outward at an oblique angle. Therefore, a calibration and compensation procedure for the laser optical axis was designed in this system. First, the beam direction was constrained by using a cage-mounted laser alignment target combined with an adjustable retaining ring lens mount, limiting the beam deviation solely in the pitch (Y) direction. Next, the reference optical path was calibrated by placing an optical height gauge 75 mm in front of the tracking head and adjusting the support rod to ensure that the laser beam passed through an 80 mm clear aperture, aligned with the vertical offset of the rotary stage center.

**Figure 5:**
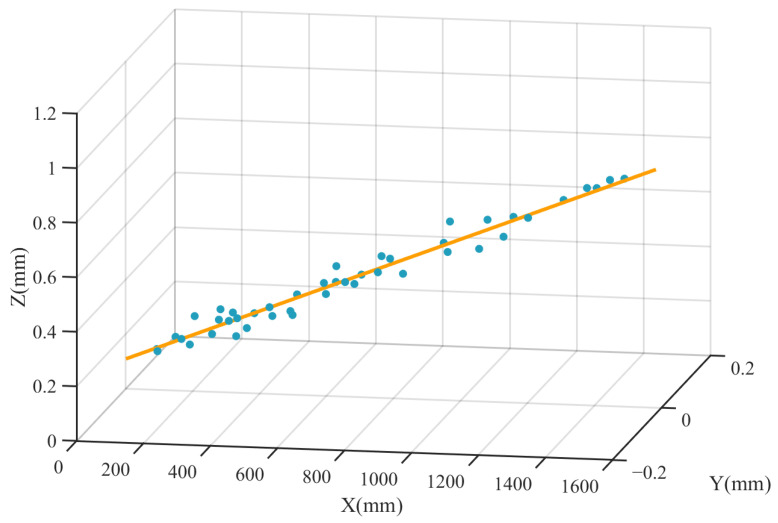
The fitting results for the laser optical axis.

Subsequently, data acquisition was performed by incrementally moving the height gauge along the laser propagation direction at 25 mm intervals while recording the beam spot height. A point cloud dataset was established with distance as the X-axis and height as the Z-axis. The experimental data obtained are shown in Figure 5. Following data collection, 30 valid data groups within the 75–800 mm range were retained. For the distal range (800–1500 mm), data points were selectively sampled. Finally, a spatial line fitting was conducted using the Singular Value Decomposition (SVD) algorithm [13] to derive the optical axis equation, ensuring coverage of 1.5 times the system’s maximum working distance requirement. The fitted laser optical axis equation is as follows:

(10) $$\left\{\begin{array}{c} y=0\\ z=0.00049x+0.11172 \end{array}\right.$$

The calibration of the distance meter optical axis requires prior measurement of its installation error. Due to the non-coincidence between the zero position of the distance meter (front surface of the housing) and the YOZ reference plane of the tracking head, a linear offset of 18.3 mm exists, defined as the primary compensation amount $d_{0}$. The calibration experiment utilized a two-dimensional detection target. First, the origin of the detection target was fixed, and the mapping coordinates of the tracking head origin on the target surface were calculated for subsequent data normalization. Then, at various mapping distances (the distance from the tracking head origin to the detection target), the two-dimensional coordinates of the distance meter’s beam spot were recorded and transformed into Y-axis and Z-axis data of the optical axis point cloud, with the mapping distance used as the X-axis. This process yielded a three-dimensional point cloud for the optical axis.

Incorporating the compensation term $d_{0}$, spatial fitting algorithms were employed to complete the optical axis calibration and correct the impact of installation errors on measurement accuracy. The point cloud data of the distance meter’s optical axis were imported into MATLAB for spatial line fitting. The resulting fitted line equation is:

(11) $$\frac{x-600}{0.99993}=\frac{y+38.18571}{-0.01088}=\frac{z+0.86}{0.00352}$$

To further improve the distance measurement accuracy, the system achieves strict coaxiality between the distance measurement beam emitted by the ADM and the main optical axis of the laser through adjustment of two reflectors. After being reflected by the reflectors, the propagation direction of the distance measurement beam remains consistent with the main optical axis of the laser over the entire working distance, with a coaxiality deviation of ≤0.01 mm. Since the distance measurement beam needs to be reflected by two reflectors, there is a fixed physical distance $d_{m}=31.22\;\mathrm{mm}$ between the reflectors. This distance will be included in the original measurement value of the rangefinder, so an additional spacing compensation needs to be added on the basis of the installation error compensation. The actual distance calculation formula is revised as:

(12) $$d_{t}=d_{c}-d_{0}-d_{m}$$

where $d_{t}$ is the actual distance, $d_{c}$ is the original measurement value of the rangefinder, $d_{0}$ is the basic compensation distance, and $d_{m}$ is the measured compensation value of the reflector spacing.

Fitted by the SVD algorithm, the optical axis equation of the system after coaxiality is:

(13) $$\left\{\begin{array}{c} y=-0.00001x+0.002\\ z=0.00049x+0.112 \end{array}\right.$$

Within the working distance of 75–1500 mm, a two-dimensional detection target is arranged every 50 mm to simultaneously record the spot coordinates of the laser and the rangefinder. The verification results show that the coaxiality deviation is ≤0.01 mm, which meets the requirements of high-precision measurement.

In this study, camera optical axis calibration and pixel-to-distance conversion factor calibration were conducted simultaneously using the camera software. The optical axis calibration was carried out in conjunction with the distance meter calibration experiment. By adjusting the lens focal length to achieve clear imaging and utilizing the center marker feature of the MVS V4.5.1 software, the mapping coordinates of the image center point on the two-dimensional detection target were recorded. Multiple data sets were obtained by moving the detection target, and spatial fitting was performed to derive the camera optical axis equation, as shown in (14):

(14) $$\frac{x-683.33333}{0.99998}=\frac{y+2.76333}{-0.00331}=\frac{z-30.21667}{-0.00216}$$

To meet the requirements for beam break recovery, it was also necessary to calibrate the pixel-to-real-distance conversion factor of the camera. Images of the detection target were captured at different radial distances, and the central 100 mm^2^ region of each image was used to calculate the pixel scale. A two-dimensional conversion curve was fitted using multiple data sets. Experimental results verified that the geometry of the camera target plane was perpendicular to the reference plane of the tracking head (rotational errors were negligible). Therefore, only the positional offset between the camera and the tracking head needed to be calibrated, eliminating the need for complex pose matrix calibration.

**Figure 6:**
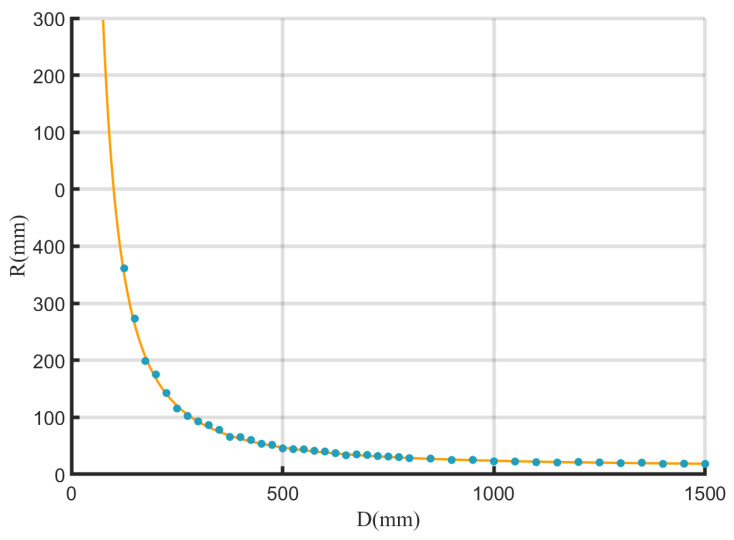
The fitting results of the camera’s length conversion function at different radial distances.

According to prior knowledge, the camera’s length conversion function typically follows a power function form. Using the Curve Fitting Toolbox in MATLAB, a two-dimensional curve fitting for the camera’s length conversion function was performed. The fitting results, presented in Figure 6, demonstrate high reliability based on metrics such as the Sum of Squared Errors (SSE), R-square coefficient, and Root Mean Square Error (RMSE). Therefore, the length conversion function R(d) of the camera at different radial distances *d* is:

(15) $$R\left(d\right)=\frac{488700}{d^{1.651}}+6.412$$

**Figure 7:**
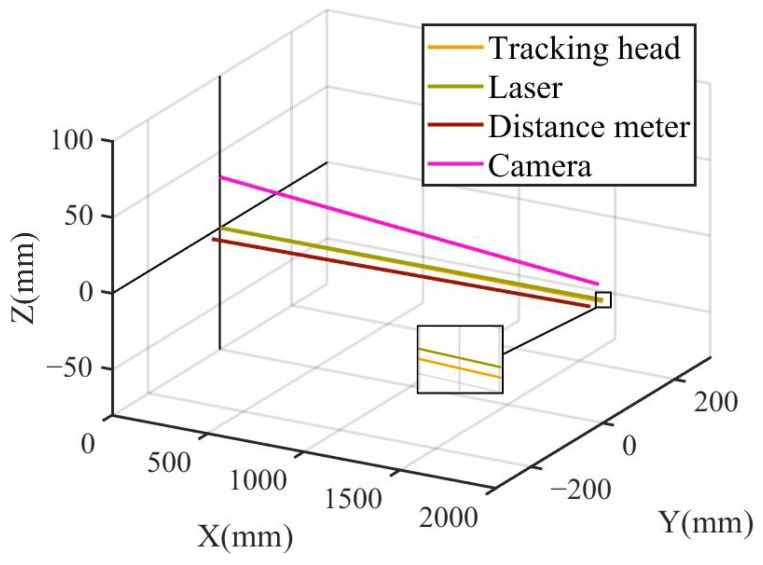
The overall optical axis model of the tracking head and its components.

The overall optical axis model of the tracking head and its components is shown in Figure 7.

### 3.3. Principle of Light Interruption and Reconnection Based on Visual Indexing

Rapid light interruption recovery is one of the prominent advantages of this study compared to traditional laser trackers. Based on the tracking head error model described in the previous section, this study designed a machine vision-based light interruption recovery control model. In this study, the state information of the target ball provided by the image processing algorithm was combined with the two parts of the tracking head optical axis model to achieve rapid light interruption recovery of the system. The corresponding control principle is as follows:

Assuming that the current measurement target is lost, the system enters a light-off state. The controller captures the video stream data from the camera and applies corresponding image processing algorithms to each frame [14,15]. After successful processing, the pixel coordinates $\left(u_{t},v_{t}\right)$ of the target ball’s center in the image and the pixel value $r_{t}$ of the target ball’s shadow ring radius are obtained. Based on the ratio to the target ball’s actual radius $r_{c}$, the pixel distance ratio of the camera image at the current radial distance is calculated:

(16) $$R_{n}=\frac{r_{t}}{r_{c}}$$

Perform the inverse transformation of Equation (15) and substitute $R_{n}$ = R(d) into it to obtain the value of the current radial distance $d_{n}$.

(17) $$d_{n}=\sqrt[{1.651}]{\frac{488700}{R_{n}-6.412}}$$

Based on the camera parameters, the pixel coordinates of the image center $C_{0}$ are (1536, 1024). Calculate the pixel difference between the center of the target ball and the $C_{0}$ point in the horizontal and pitch directions, and then combine it with Rn to convert it into a real distance value:

(18) $$D_{Ht-c}=\frac{u_{t}-1536}{R_{n}}$$

(19) $$D_{Vt-c}=\frac{v_{t}-1024}{R_{n}}$$

In this equation, $u_{t}$ and $v_{t}$ are the horizontal pixel coordinate and pitch pixel coordinate of the target ball’s center. $D_{Ht-c}$ represents the true distance of the position of the target ball relative to the optical axis of the camera in the horizontal direction, and $D_{Vt-c}$ represents the true distance of the target ball position relative to the camera optical axis in the pitch direction.

Substitute the radial distance $d_{n}$ as the value of the X axis into the camera optical axis model Equation (14), to obtain the true distance differences $D_{Hc-0}$ and $D_{Vc-0}$ between the camera optical axis and the main optical axis of the tracking head in the horizontal and pitch directions, respectively, at the current radial distance $d_{n}$:

(20) $$D_{Hc-o}=-0.0033\;d_{n}-0.5014$$

(21) $$D_{Vc-o}=-0.0022\;d_{n}+31.6927$$

Calculate the true distances $D_{Ht-0}$ and $D_{Vt-0}$ of the target ball center position relative to the main optical axis of the tracking head in the horizontal and pitch directions:

(22) $$D_{Ht-o}=D_{Ht-c}+D_{Hc-o}$$

(23) $$D_{Vt-o}=D_{Vt-c}+D_{Vc-o}$$

Finally, use the inverse tangent function to calculate the deflection angles $Rad_{H}$ and $Rad_{V}$ of the instrument reference point (the origin of the tracking head coordinate system) from the center of the target ball in the horizontal and pitch directions:

(24) $${\mathrm{Rad}}_{H}=\tan^{-1}\left(\frac{D_{Ht-o}}{d_{n}}\right)$$

(25) $${\mathrm{Rad}}_{V}=\tan^{-1}\left(\frac{D_{Vt-o}}{d_{n}}\right)$$

**Figure 8:**
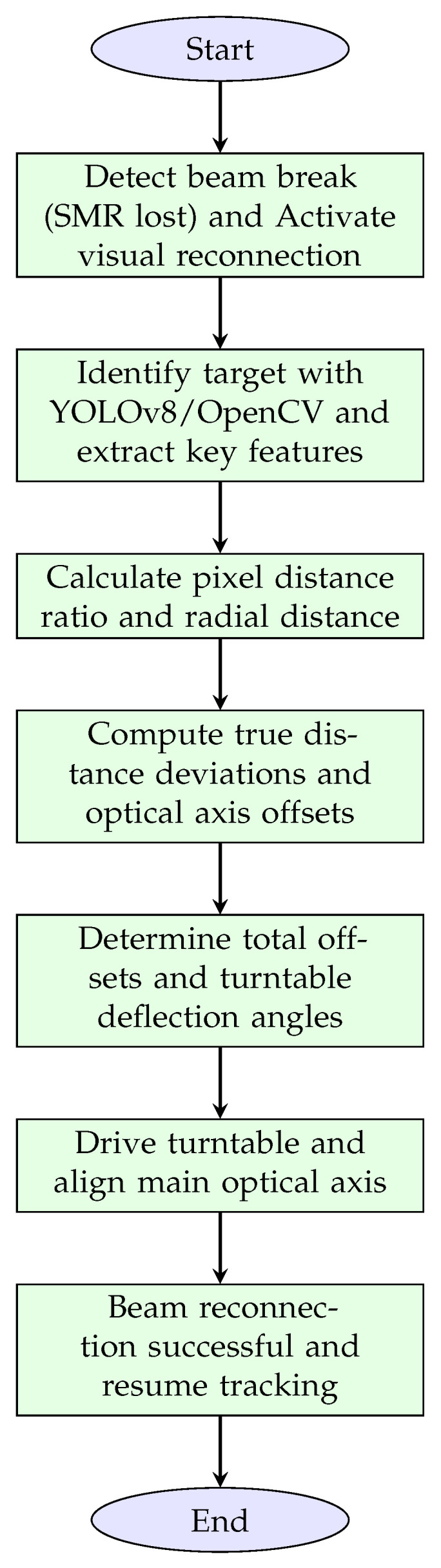
Highly concise flowchart of the visual indexing-based beam reconnection algorithm.

After all the above calculations are completed, the system will drive the two-axis turntable mechanism to rotate toward the target ball based on the values of $Rad_{H}$ and $Rad_{V}$, thereby enabling the main optical axis of the tracking head to successfully align with the center of the target ball and complete the instrument’s light interruption recovery. To systematically encapsulate the closed-loop control logic of the visual indexing-based beam reconnection algorithm detailed earlier, Figure 8 presents a highly concise flowchart. This diagram integrates the core operational chain from beam-break detection to successful beam reconnection, encompassing target recognition, spatial parameter calculation, offset analysis, and turntable adjustment. Each node reflects the key functional steps of the algorithm, directly mapping to the systematic analysis and computational processes outlined in the preceding sections. By visualizing the sequential workflow, the flowchart offers an intuitive overview of how the system achieves rapid, initialization-free beam recovery.

## 4. System Implementation and Experiments

### 4.1. Hardware Selection and Assembly

The system employs a biaxial rotary stage as the driving mechanism for the tracking head. The core performance parameters are listed in Table 4. The rotary stage utilizes a worm gear drive and a 100 W servo motor, supplemented with a LAMOTION circular encoder to achieve an angular resolution of $0.019^{\prime \prime }$. The system adopts triple closed-loop control, incorporating current, velocity, and position loops. Communication with the host computer is established via an RS232 serial interface, which supports secondary development through the Software Development Kit (SDK).

The tracking head (127 × 113 × 128 mm, 1.2 kg) is connected to the rotary stage platform via an optical support rod. The support rod is adjusted to align the reference point with the 80 mm customized vertical offset from the stage platform, and the optical path is calibrated using an optical scale. The integrated vision-based laser tracker satisfies both laser interferometric distance measurement and dynamic tracking requirements.

### 4.2. Software Design

**Figure 9:**
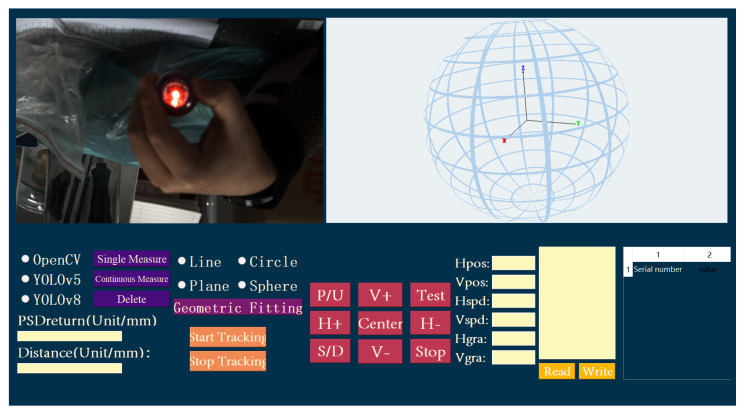
The display interface of the host computer software in this system.

The upper computer control software was developed using C++17 and the Qt5.14.2 framework, as shown in Figure 9. It integrates OpenGL for 3D rendering and the Point Cloud Library (PCL1.14.0) for point cloud processing, enabling precise control and data management for the laser tracker. The core software functions include instrument control, dynamic tracking, data visualization, and algorithm integration. Pan-tilt motion control (at a speed of 10°/s), emergency stop, and zero-reset operations are implemented via interface buttons, with command transmission managed through the Qt signal-slot mechanism and the rotary stage control library.

The real-time monitoring module refreshes rotary stage angles (with a grating closed-loop resolution of $0.019^{\prime \prime }$), speed, PSD spot coordinates (tolerance of 0.1 mm), and distance meter correction values at 50 Hz. Dynamic system status is indicated using color-coded lights: blue for running, red for beam loss, orange for beam alignment, and green for target acquisition. The 3D visualization module, based on OpenGL, constructs the measurement coordinate system, dynamically rendering a magnetic field line model (with a 1 m radius representing actual distance) and measurement points (represented as blue-black spheres). It supports mouse-based interaction for viewpoint adjustment and zooming, offering intuitive visualization of spatial data distribution.

Dynamic tracking functionality is executed in a separate thread, integrating PSD feedback and vision-based algorithms (supporting YOLOv5/v8 and OpenCV) for beam break recovery. When spot displacement exceeds the threshold, the rotary stage triggers closed-loop adjustment. The data recording module supports single or continuous measurements (at 1 s intervals), converting polar coordinates into 3D coordinates for storage while providing invalid data filtering.

Point cloud processing leverages the PCL library for spatial line and plane fitting as well as custom model fitting (e.g., arcs, spheres) using least-squares methods. Fitting results are overlaid onto the OpenGL visualization. The camera module interfaces with the Hikvision SDK for image stream callbacks, performs distortion correction, and utilizes OpenCV or YOLO (converted to the cross-platform ONNX format) for real-time target sphere detection with overlaid visualization of detection results. The parameter configuration interface supports read/write operations for 15 parameters, including rotary stage acceleration and camera exposure settings, automatically detecting serial ports and initializing devices.

Communication between the system and the rotary stage control unit is established via RS232 serial communication. Triple closed-loop control (current, velocity, position) ensures high-precision dynamic response, and the grating closed-loop enhances angular resolution to $0.019^{\prime \prime }$. The main control thread coordinates PSD, distance meter, and vision subsystems, ensuring real-time performance and system stability to meet the high-precision measurement and rapid recovery demands of the laser tracker in complex scenarios.

### 4.3. The Workflow of the Laser Tracking System

**Figure 10:**
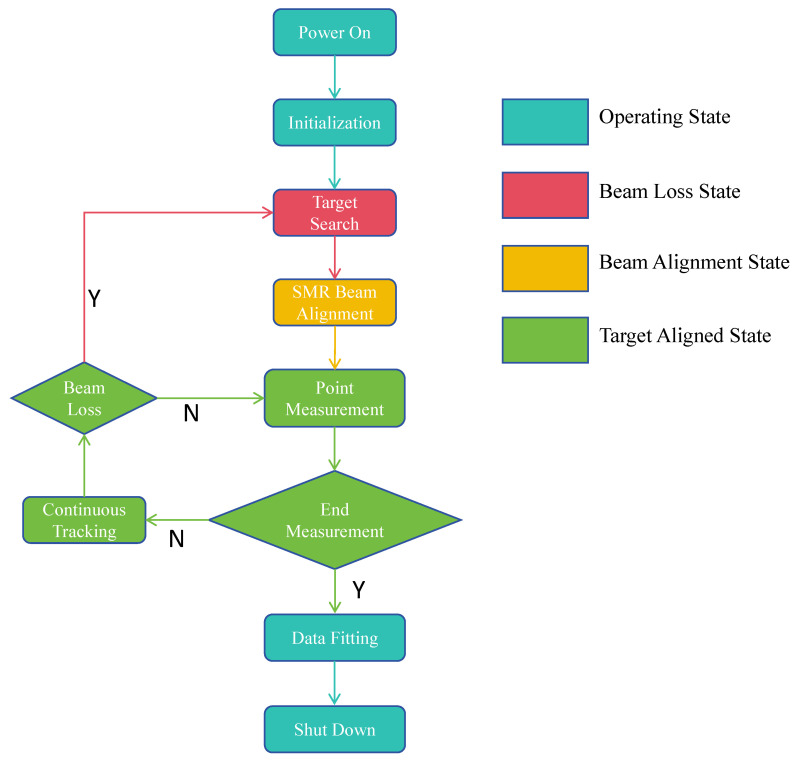
Workflow diagram of the proposed system.

Similar to traditional laser trackers, this system is also divided into multiple operating states. The specific work tasks and workflow are shown in Figure 10. After receiving the power-on command, the system first performs serial port information checks on all sensors. Once confirmed correct, it connects all sensors to complete the startup process, and the system enters the basic operating state from this point. During startup, system initialization is also carried out, which mainly includes setting initial parameters of each sensor, checking optical axis offset, etc. After all parameter settings are completed, the tracking and measurement function can be activated: first, place the target SMR at the point to be measured—at this moment, the system is in the beam loss state, and it executes the target recognition algorithm for the SMR on the camera image. Then, relying on the beam break recovery control model, it deduces the world coordinates of the SMR from the pixel coordinates of the SMR’s center. After obtaining these coordinates, the system controls the rotary stage to turn toward the target. When the rotary stage moves to a position where the laser beam can enter the interior of the SMR, the PSD outputs the offset of the reflected light spot, and the system enters the beam alignment state at this time. In the beam alignment state, the current spherical coordinate position of the SMR is calculated based on the output value of the PSD sensor and the distance measured by the rangefinder, and the rotary stage will continue to rotate until the PSD output value is zero—at this point, the system enters the target-aligned state. Coordinate measurement can be performed in the target-aligned state; after measurement, move the target SMR to another measurement point and repeat the measurement until all points to be measured are completed. If the system enters the beam loss state during measurement (due to reasons such as the SMR moving too fast or the optical path being blocked), the system will re-execute the target search task until it enters the target-aligned state again. If all points to be measured have been completed, the system will perform point cloud data fitting according to the selected fitting target, and after fitting, output basic geometric parameters as reference results. At the end of the system’s operation, position re-measurement and data export can be performed optionally; if not needed, all processes are terminated and the system returns to the basic operating state.

### 4.4. Precision Testing Experiment

A relative error test was conducted on the independently developed off-axis laser tracker. Due to the off-axis measurement approach, where the laser and distance measurement axes are separated, traditional error models were not directly applicable. Therefore, the system’s performance was evaluated by comparing measurement data from the proposed system (experimental group) and a commercial FARO laser tracker (reference group).

A Thorlabs high-precision two-axis electric displacement stage (accuracy: 0.1 μm) was used to drive the SMR target sphere along the Y and Z axes (horizontal range: 0–8 mm, vertical range: 0–15 mm, with 1 mm steps). A total of 144 sets of 3D coordinate data were simultaneously recorded from both the proposed system and the FARO tracker (preheated for 30 min) [16].

**Figure 11:**
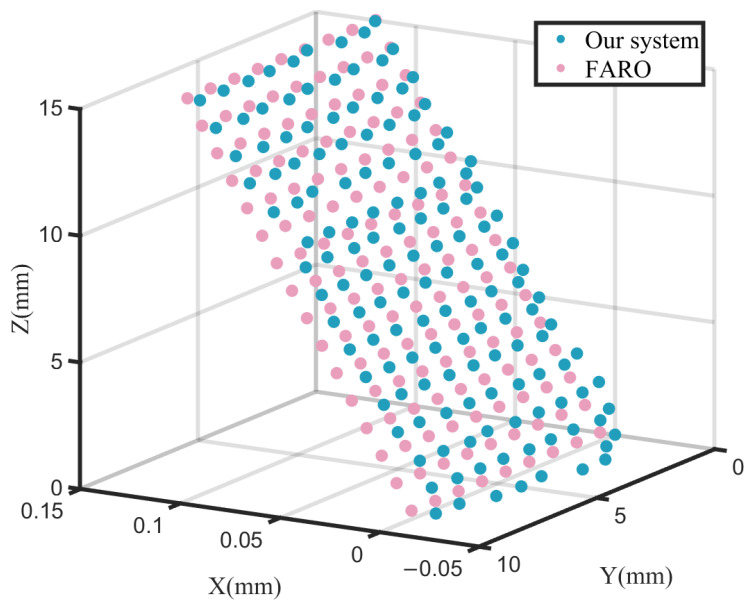
Three-dimensional visualization of the two experimental datasets.

The datasets from the FARO tracker and the proposed system were designated as the reference and experimental groups, respectively. Since both datasets were measured within their respective coordinate systems, the reference group data were rescaled and reprojected onto the experimental system’s coordinate framework for coordinate-aligned comparative analysis. The two point clouds were visually displayed in 3D using MATLAB, as shown in Figure 11.

By assuming parallelism between the XOY planes of the two systems and retaining only rotation around the Z-axis and origin translation parameters, the reference group data were projected onto the coordinate system of the experimental group. Based on the experimental data, a statistical analysis was conducted to evaluate the mean error (ME), maximum error (R), standard deviation (SD), and root mean square error (RMSE) of the proposed system relative to the FARO laser tracker. Relative errors between corresponding measurement points from both datasets were calculated for the X, Y, and Z measurement directions, as well as for the spatial Euclidean distance. The results are presented in Table 5.

The results indicate that the mean error in the X direction was −0.025 mm, with an RMSE of 0.030 mm, primarily influenced by the stability of the distance meter. The RMSEs in the Y and Z directions reached 0.150 mm and 0.129 mm, respectively, reflecting combined limitations in distance meter accuracy and rotary stage stability. The spatial distance exhibited a mean error of 0.191 mm and a maximum error of 0.945 mm, revealing deficiencies in the mechanical repeatability of the system.

Additionally, the experiment observed a systematic X-axis displacement of approximately 0.009 mm per millimeter during Y/Z axis movement of the target sphere. This phenomenon is attributed to the assembly inclination of the displacement stage. However, as the reference group data inherently compensated for this effect, this error does not significantly impact the performance evaluation of the proposed system.

The primary error sources of the system were identified as angular error, distance measurement error, tracking error, and assembly error. Firstly, angular error mainly arises from the angle measurement error of the rotary encoder, as well as orthogonality deviation between the two axes of the rotary stage and mechanical installation errors, all of which further affect attitude calculation accuracy. Based on the rotary stage repeatability specification of 0.003°, and applying small-angle approximation, the angular error-induced spatial deviation at a 1 m measurement range was approximately 0.052 mm.

Secondly, distance measurement error mainly originates from the random noise and systematic bias of the distance meter itself. According to the specifications of the selected distance meter, the distance measurement error within a 1 m range was approximately 0.06 mm. Thirdly, tracking error refers to positional deviations caused by servo lag, dynamic dead zones, and control response delays during dynamic target tracking. Finally, assembly error primarily includes orthogonality deviation between the two axes, installation errors of optical components, and central axis misalignment. These assembly errors lead to discrepancies between the actual measurement direction and the ideal geometric model, thereby affecting measurement accuracy.

According to the measurement results in Table 5, the spatial RMSE of the system was 0.189 mm. Considering that the combined contribution of angular and distance measurement errors was 0.112 mm, the remaining error of approximately 0.077 mm can be primarily attributed to the combined effects of tracking error and assembly error.

Although the system achieved single-axis measurement accuracy at the 0.05 mm level within a 1.0 m measurement range, spatial error was evidently constrained by the stability of the rotary stage control. The observed high maximum error and RMSE indicate that further optimization of the mechanical structure and control algorithms is necessary. The experiments validated the baseline measurement capability of the prototype system, providing essential data support for future accuracy enhancement and functional improvements. To clarify the quantitative improvement effect of reducing or compensating key error sources on the total measurement error, a quantitative analysis is conducted based on the error decomposition results (Table 5) and the independent contribution ratio of each error source, las follows:

1. Quantitative improvement potential of angular error. The current angular error (caused by encoder resolution, turntable axis orthogonality deviation, and mechanical installation errors) results in a spatial deviation of approximately 0.052 mm at a measurement distance of 1 m, accounting for 27.5% of the total error (0.052/0.189). By adopting a higher-resolution encoder (improving the repeatability from the existing 0.003° to 0.001°) and correcting the axis deviation through an orthogonality error compensation algorithm, the spatial deviation caused by angular error can be reduced to 0.017 mm. This single reduction contributes approximately 0.035 mm, corresponding to an 18.5% decrease in the total measurement error.
2. Quantitative improvement potential of distance measurement error. The inherent error of the distance measurement module (random noise + systematic deviation) is approximately 0.06 mm, accounting for 31.7% of the total error (0.06/0.189). By introducing a temperature compensation algorithm (correcting the impact of ambient temperature on distance measurement accuracy) and moving average filtering to handle random noise, the distance measurement error can be reduced to 0.03 mm. This single reduction contributes approximately 0.03 mm, corresponding to a 15.9% decrease in the total measurement error.
3. Quantitative improvement potential of assembly error. The current contribution of assembly error (including axis orthogonality deviation, optical component installation errors, and central axis misalignment) is approximately 0.077 mm, accounting for 40.8% of the total error (0.077/0.189). By improving mechanical processing precision (optimizing the axis orthogonality deviation from the current 0.01 mm to 0.005 mm) and performing precise calibration of the optical axis using a laser interferometer, the assembly error can be reduced to 0.01 mm. This single reduction contributes approximately 0.067 mm, corresponding to a 35.4% decrease in the total measurement error.

Combining the optimal reduction schemes for the three types of errors mentioned above, the total measurement error of the system can be reduced from the current spatial RMSE = 0.189 mm to approximately 0.085 mm, with a relative improvement of 55.0%. At this point, the relative positioning accuracy of the system at the maximum working distance of 1.5 m can be better than 0.1 mm, further meeting the requirements of high-precision industrial measurement scenarios. The above quantitative analysis is based on the assumption of independent error effects; in practical applications, the error coupling effect needs to be considered, and the final accuracy improvement is expected to be between 50% and 55%.

## 5. Conclusions

This study addresses the technical pain points of traditional laser trackers, such as low beam break recovery efficiency and strict dependence on optical coaxiality and mechanical processing precision. A machine vision-based laser 2D tracking system with rapid beam break recovery capability is proposed, and the system architecture design, multi-dimensional error calibration method research, prototype development, and experimental verification are completed. The main research conclusions are as follows:

1. An off-axis optical splitting design scheme is proposed, where the vision recognition optical path and the distance measurement optical path are independently configured. The coordinated cooperation of the two optical axes is realized through a cube beam splitter, which effectively reduces the system’s dependence on high-precision optical coupling and mechanical assembly. Meanwhile, the system miniaturization (300 mm × 300 mm × 310 mm) and low-cost development (prototype cost of approximately $25,000) are achieved, providing a cost-effective solution for short-distance high-precision measurement scenarios.

2. A visual indexing-based beam break recovery mechanism is constructed, forming a closed-loop process of “beam break detection → target ball visual positioning → pixel-distance conversion → spatial offset calculation → turntable attitude adjustment”. Rapid beam reconnection can be completed without re-initialization, which solves the problem of low recovery efficiency of traditional laser trackers after beam break and improves the continuity of dynamic measurement.

3. A multi-dimensional error calibration system is established to provide key support for the high-precision measurement of the system: (1) Zhang’s calibration method for monocular cameras is adopted, which can simultaneously solve intrinsic and extrinsic parameters using a planar checkerboard target. It effectively corrects lens radial distortion (0.0036) and tangential distortion (−0.0013) without the need for high-precision 3D calibration components, reducing calibration costs and operational complexity. (2) A tracking head optical axis calibration method based on the Singular Value Decomposition (SVD) algorithm is proposed. By fitting the optical axis point cloud data of the laser, rangefinder, and camera, the installation errors of optical components (e.g., 18.3 mm linear offset of the rangefinder) and beam tilt deviations are quantified and compensated. After calibration, the coaxiality deviation of the three optical axes is ≤0.01 mm, which significantly reduces the inherent system error. (3) A power function-based pixel-distance conversion model is established. Experimental verification shows excellent fitting reliability, and the radial distance can be inversely derived only through the ratio of the pixel radius of the target ball’s shadow ring to the actual radius, providing a fast and accurate distance estimation basis for beam break recovery.

4. Experimental verification and error analysis indicate that the system achieves a spatial Root Mean Square Error (RMSE) of 0.189 mm and a relative positioning accuracy better than 0.2 mm at the maximum working distance of 1.5 m, meeting the requirements of short-distance high-precision measurement. Meanwhile, it is clarified that the tracking head mechanical system, rotary encoder, and distance measurement module are the core factors determining the stable and accurate operation of the device. Among them, the assembly accuracy of the tracking head mechanical structure (e.g., axis orthogonality deviation, optical component installation coaxiality), the angle measurement accuracy of the rotary encoder, and the measurement accuracy of the distance measurement module together constitute the main sources of the system’s total error, and are also the key links restricting the improvement of the system’s measurement accuracy.

Future research will focus on three optimization directions: first, improving optical coupling efficiency and operational stability under complex environmental conditions (e.g., reducing the impact of ambient light interference); second, enhancing the rigidity and assembly precision of the tracking head’s mechanical structure to reduce axis deviation and optical component installation errors; third, adopting high-resolution encoders and developing targeted angular error compensation algorithms to further weaken the constraints of mechanical defects and encoder performance on measurement accuracy, providing technical support for the iterative upgrading of system performance. 

## Figures and Tables

**Table 1:** Characteristic comparison of the proposed system and commercial laser trackers.

Characteristic Indicators	Proposed System	API T3	Leica AT960
Total System Weight	≤10 kg (simplified structure)	23 kg (with controller)	21 kg (integrated host)
Core Optical Architecture	Dual-mirror coaxial design (adapted for short-distance)	Built-in multi-optical components (general long-distance)	Built-in multi-optical components (general long-distance)
Anti-interference Adaptation Scenarios	Short-distance, indoor complex light environment (no external shading required)	Long-distance, stable lab environment (temperature control dependent)	Long-distance, standard working conditions (temperature control dependent)
Measurement Distance Range	0∼1.5 m (short-distance dedicated)	0∼60 m (long-distance general)	0∼60 m (long-distance general)
Total Calibration Time (including preheating)	≤5 min (rapid assembly)	15 min (13 min preheating + 2 min calibration)	≥12 min (professional operation required)
Main Application Scenarios	Short-distance high-precision assembly, indoor small-space measurement	Large-size workpiece inspection, long-distance positioning	Large equipment calibration, ultra-long-distance measurement

**Table 2:** Component information list of the tracking head.

Figure Label	Component Category	Component Model	Origin (City, Country)	Quantity
1	Cube Beam Splitter	M2-BS1	Guangzhou, China	1
2	Industrial Camera	MV-CE060-10UC	Hangzhou, China	1
3	Rangefinder	L2	Shenzhen, China	1
4	PSD Sensor	DRX-PSD500	Shenzhen, China	1
5	Laser	HW650DMGX100-1660BD	Xi’an, China	1
6	Industrial Lens	MVL-HF1624M-10MP	Hangzhou, China	1
7	PSD Amplifier Circuit Board	DRX-2DPSD-OA11	Shenzhen, China	1
8	Adjustable Fixing Frame	CRD-2X	Guangzhou, China	1
9	Cage Plate Adapter	LPCM-C	Guangzhou, China	1
10	Cage Coaxial Rod	PCM-S19	Guangzhou, China	4
10	Cage Coaxial Rod	PCM-S38	Guangzhou, China	4
10	Cage Coaxial Rod	PCM-S76	Guangzhou, China	4
11	Pressure Ring Lens Holder	CSJ-25	Guangzhou, China	2
12	Laser Mounting Hole Frame	POL-12	Guangzhou, China	2
13	Lens Light Shield Tube	CSA-45	Guangzhou, China	2
13	Lens Light Shield Tube	CSA-08	Guangzhou, China	1
13	Lens Light Shield Tube	CSA-08T	Guangzhou, China	1
14	Narrow Band Filter	NP650	Guangzhou, China	3
15	Telescopic Optical Support Rod	CAT57-S	Guangzhou, China	1
16	Coaxial Light Shield Tube	Customized	Guangzhou, China	1

**Table 3:** Camera Parameters.

Parameters	Value
Image Size	2048, 3072
Radial Distortion	0.0036, 0.4924
Tangential Distortion	−0.0013, 0.0040141

**Table 4:** Core performance specifications of the selected biaxial rotary stage.

Structural Parameters	
Angle Range	Azimuth $\pm 180^{\circ }$, Elevation $\pm 90^{\circ }$
Platform Size	165 × 80 mm
Transmission Ratio	180:1
Drive Mechanism	Worm gear and worm shaft mechanism
Motor	100 W servo motor
Central Load Capacity	10 kg
**Accuracy Parameters**	
Resolution	$0.0005^{{}^{\circ}}$ = $1.8^{\prime \prime }$ (with 20 subdivisions)
Closed-Loop Mechanism	Grating closed-loop (resolution: $0.0001125^{{}^{\circ}}$)
Maximum Speed	$50^{{}^{\circ}}$/s
Repeatability	$0.003^{{}^{\circ}}$
End Jump Accuracy	10 μm

**Table 5:** Measurement errors in different dimensions.

Measurement Dimension	Mean Error (ME)	Maximum Error (R)	Standard Deviation (SD)	Root Mean Square Error (RMSE)
X Direction	−0.025	0.083	0.030	0.030
Y Direction	0.044	0.937	0.147	0.150
Z Direction	0.034	0.761	0.126	0.129
Spatial Distance	0.191	0.945	0.099	0.189

## Data Availability

The data presented in this study are available on request from the corresponding author.

## Abbreviations

SMR	Spherically Mounted Retroreflector
PSD	Position Sensitive Detector
ADM	Absolute Distance Meter
SVD	Singular Value Decomposition
RMSE	Root Mean Square Error
SD	Standard Deviation
ME	Mean Error
